# Novel surgical approach to neoplastic lesions in the distal part of the urethra: A pilot cadaver study comparing open and hybrid techniques

**DOI:** 10.1111/vco.12798

**Published:** 2022-01-07

**Authors:** Przemysław Prządka, Bartłomiej Liszka, Agnieszka Antończyk, Ludwika Gąsior, Zdzisław Kiełbowicz

**Affiliations:** ^1^ Department and Clinic of Surgery, Faculty of Veterinary Medicine Wroclaw University of Environmental and Life Sciences Wroclaw Poland; ^2^ Department of Internal Diseases and Diagnosis, Faculty of Veterinary Medicine and Animal Sciences Poznan University of Life Sciences Poznan Poland

**Keywords:** dog, laparoscopy, surgery, urethra, urethrostomy

## Abstract

Tumours of the distal urethra in female dogs are often difficult to treat, and the surgical methods described thus far have technical limitations. This study aimed to present a novel approach to the surgical treatment of distal urethral tumours. This study used dog cadavers to evaluate the technical feasibility of surgically removing neoplastic lesions in the distal urethra and compared surgical outcomes of open surgery with those of hybrid surgery (combination of laparoscopy and open surgery). Open intact, open spayed, hybrid intact, and hybrid spayed dog cadaver groups underwent surgery (*n* = 6 per group). The novel surgical method was based on vulvovaginectomy (ovariohysterectomy in intact dogs), resection of the distal part of the urethra, and pre‐pubic urethrostomy. Outcomes of interest included technical feasibility of each procedure, using both surgical techniques, wound length, time required to complete the procedure, and the incidence of intraoperative ureter and rectum injuries. Surgical technique and reproductive status affected operating time. Technique choice affected wound length; the surgical wound was longer in the open group than in the hybrid group. Macroscopic evaluation of the rectum did not reveal any damage to the wall. There was no evidence of ureter leakage or obstruction in any case. The present findings suggest that both open and hybrid surgery can be used to treat distal urethral tumours.

## 1 INTRODUCTION

Neoplasms of the urinary system in dogs account for <0.5% to 2% of all tumours in this species, depending on their origin.^1^, ^2^ Lower urinary tract tumours account for <1% of tumours in dogs.^2^ Most of these tumours are malignant and epithelial in origin, including transitional cell carcinoma, which is the most common tumour type and which often affects female dogs.^1^, ^2^, ^3^

Extensive tumours of the distal urethra in female dogs rarely allow for complete removal and simultaneous reattachment of the severed urethra using an end‐to‐end technique. Such cases may require pre‐pubic urethrostomy, vaginourethroplasty or conservative management.^4^, ^5^ The technical possibilities of vaginourethroplasty are limited,^4^, ^5^ while pre‐pubic urethrostomy alone is a palliative form of treatment. Other palliative approaches include cystostomy,^6^, ^7^ urethral stenting^8^, ^9^, ^10^ and transurethral resection.^11^

The present study aimed to propose a novel surgical technique for the treatment of proliferative changes in the distal part of the urethra in cases that require an oncological margin. This study aimed to develop and evaluate a method for radical lesion removal (with the theoretical possibility of maintaining a healthy tissue margin) while maintaining an undisturbed urine outflow. The procedure was performed on cadavers using both open and hybrid (combination of laparoscopy and open surgery) approaches; the characteristics and outcomes of these procedures were compared.

## 2 MATERIALS AND METHODS

This study was performed on dog cadavers and was approved by the relevant ethics committee (060/2020). The study procedures were performed within 24–72 h after the animals' natural death or euthanasia for reasons unrelated to the experiment. All procedures were performed by the same operators with experience in both laparoscopic and open surgery.

The study cadavers included 12 previously castrated female dogs, spayed, and 12 non‐castrated female dogs, intact (Tables 1 and 2). The animals were divided into four groups: open intact, including intact female dogs that underwent open surgery (*n* = 6); open spayed, including spayed female dogs that underwent open surgery (*n* = 6); hybrid intact, including intact female dogs that underwent hybrid surgery (*n* = 6); hybrid spayed, including spayed female dogs that underwent hybrid surgery (*n* = 6).

**TABLE 1:** Characteristics of the dogs included in the open surgery group

Open surgery						
				Wound length (cm)		
				Castrated		
Number	Breed	Age (year)	Weight (kg)	No	Yes	Time (minutes)
1	Siberian Husky	6	21.5	24	X	71
2	Golden retriever	7.5	28	31.5	X	78
3	Golden retriever	8	25	28.5	X	83
4	Crossbreed	5	29.5	31	X	76
5	French Bulldog	7.5	12	20.5	X	64
6	Crossbreed	4	11	21	X	72
7	German Shepherd	8.5	38	X	24	67
8	Golden retriever	7.5	30	X	22.5	63
9	Siberian Husky	6.5	23	X	17.5	59
10	Irish setter	7	28	X	20.5	54
11	Crossbreed	9	35	X	23	66
12	German Shepherd	9	32	X	23	61

**TABLE 2:** Characteristics of the dogs included in the hybrid surgery group

Hybrid surgery								
				Wound length (cm)				
				Castrated				
Number	Breed	Age (year)		No		Yes		Time (minutes)
			Weight (kg)	Vulva	Trocars (sum)	Vulva	Trocars (sum)	
13	French Bulldog	8	13	6.5	1.5	X	X	88
14	Crossbreed	7	15	5.5	1.5	X	X	96
15	German Shepherd	8	34	8.5	1.5	X	X	78
16	Labrador retriever	5.5	30	8	1.5	X	X	101
17	Crossbreed	9.5	23.5	8	1.5	X	X	85
18	Labrador retriever	7	29	8.5	1.5	X	X	98
19	Crossbreed	6.5	25	X	X	7.5	1.5	63
20	Golden retriever	9	26	X	X	7	1.5	69
21	Crossbreed	5	26	X	X	7.5	1.5	75
22	English bulldog	8	18	X	X	6.5	1.5	71
23	Crossbreed	4.5	11.5	X	X	6	1.5	64
24	German Shepherd	7.5	45	X	X	9	1.5	72
13	French Bulldog	8	13	X	X	7.5	1.5	88

The cadavers were placed in dorsal recumbency on the operating table, with the pelvic region placed on the edge of the operating table to facilitate the preparation of the vulva and vagina during surgery. The duration of each procedure was measured, and the ureter and rectal wall were examined postoperatively for evidence of damage.

### 2.1 Open surgery

**FIGURE 1:**
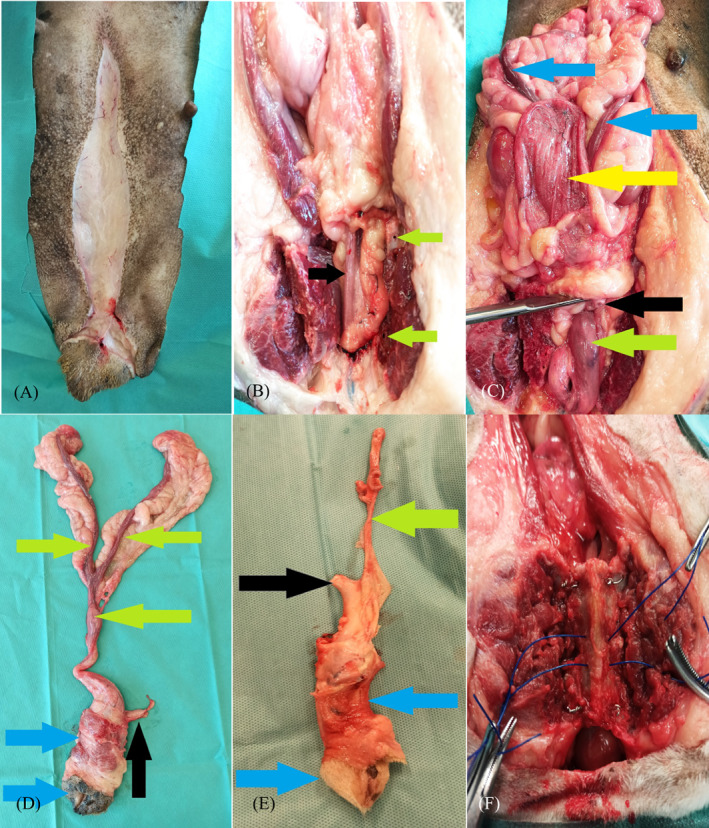
(A) Intraoperative appearance of the abdominal wound in dogs undergoing open surgery. (B) Abdominal access achieved after pelvic symphysis osteotomy. Holes drilled in the bones for subsequent osteosynthesis (green arrows) and the urethral route (black arrows) is visible. (C) Surgical field observed after transversely severing the urethra, including the severed proximal part of the urethra (black arrow), cervix (green arrow), uterine horns exposed after ligation of ovarian peduncles and dissection of broad ligaments of the uterus (blue arrows), and bladder (yellow arrow). (D) Urogenital organs removed from the open intact group, including the distal part of the urethra (black arrow), body with horns of the uterus (green arrows), and vulva with vaginal vestibule (blue arrows). (E) Urogenital organs removed from the open spayed group, including the distal part of the urethra (black arrow), uterine stump (green arrow), and vulva with vaginal vestibule (blue arrows). (F) Intraoperative image obtained during pelvic symphysis osteosynthesis in the open group

Median laparotomy involved a cutaneous incision made from the umbilical region (open intact group) or from a half‐way point between the umbilicus and pubic symphysis (open spayed group) up to the perineum, surrounding the vulva with an elliptical incision (Figure 1A). The pelvic bone osteotomy was performed according to the technique described by Allen and Crowell.^12^ The space between the right and left adductor muscles was cut and the adductor muscles were elevated subperiosteally from the pubis and ischium to expose the obturator nerves and approximately half of the obturator foramina. The pre‐pubic tendon was transected along the left side of the pubis to the proposed pubic osteotomy site. Holes were drilled in the pubis and ischium on both sides of the four proposed osteotomy sites and craniocaudally along the left pubis. The pubic and ischial osteotomies were performed using an oscillating saw (Conmed, Conmed Corporation, NY, USA). The internal obturator muscle was elevated subperiosteally from the left pubis and ischium, allowing retraction of the central bony plate to the right. After obtaining access to the pelvic cavity, the main part of the procedure was performed (Figure 1B). The ovarian pedicles (open intact group) and the broad ligaments of the uterus were ligated and dissected up to the uterine body (absorbable monofilament material, Monosyn 0, B Braun, Rubi, Spain). The distal part of the urethra was dissected and cut transversely in the middle of its length, followed by dissection of the uterine body, vagina and distal urethra (Figure 1C). The vaginal branches of the vaginal artery and vein, as well as the uterine arteries and their branches to the vagina were coagulated. The dissection was performed caudally around the vagina by transecting the ischiocavernosus and ischiourethralis muscles and passing caudally between the paired levator ani muscles to the level of the cervix. The constrictor vestibule and constrictor vulvae muscles were dissected from the vestibule. The dorsal labial branches of the ventral perineal artery were coagulated. The superficial tissues were sharply dissected from the labia and vestibule. All these organs were removed after dissection, including the previously dissected uterus and ovaries (Figure 1D). In the open spayed group, the procedure was initiated with a transverse cut of the urethra in the middle of its length, followed by organ dissection starting from the uterine stump and distal part of the urethra (Figure 1E). The rest of the procedure followed a protocol comparable to that followed in the open intact group.

**FIGURE 2:**
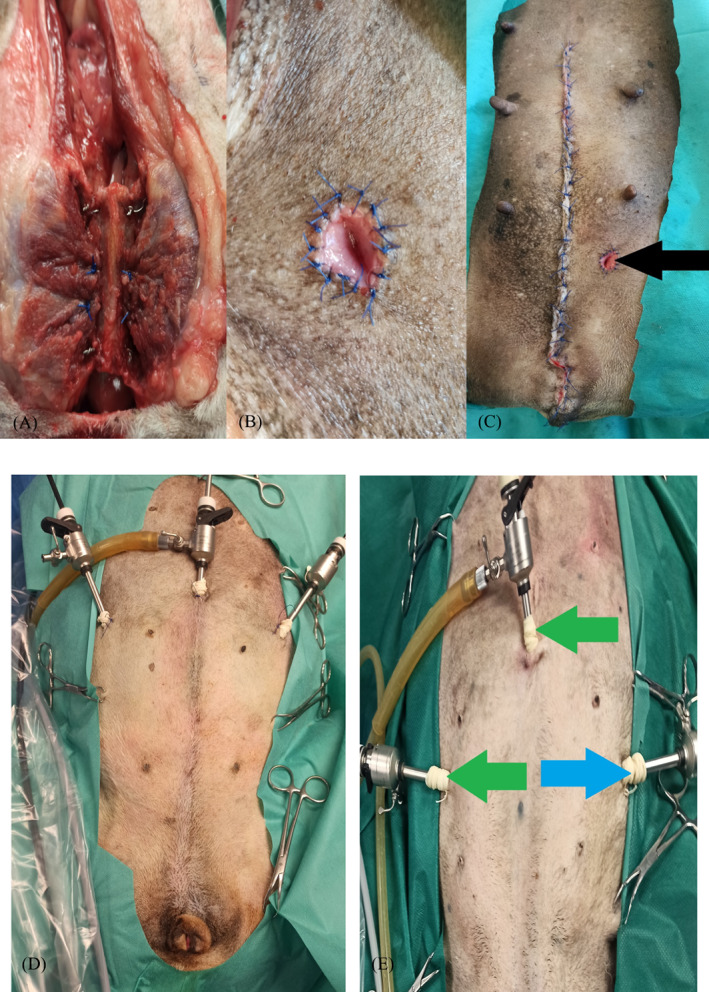
(A) Intraoperative image obtained after pelvic symphysis anastomosis in the open groups. (B) Intraoperative image of paramedial pre‐pubic urethrostomy. (C) Postoperative wound in the open intact group, including the site of paramedial urethrostomy (black arrow). (D) Trocar system in the hybrid group. (E) Trocar system during removal of the right ovary in the hybrid intact group, showing the optical trocar (blue arrow), and working trocars (green arrows)

The final part of the procedure in the open intact and open spayed groups involved osteosynthesis of the previously performed osteotomy. Strands of 18‐ to 20‐gauge stainless steel orthopaedic wires were placed through the previously drilled holes at each osteotomy site. Strands of 2–0 polypropylene were preplaced through the line of holes in the left pubis and ischium, through the left internal obturator muscle, and back through the adjacent holes in the pubis or ischium, before replacing the bony plate. The bony plate was reduced, and the preplaced wires and sutures were secured. The adductor muscles were sutured with horizontal mattress sutures using absorbable monofilament material (Monosyn 0, B Braun, Rubi, Spain) (Figures 1F and 2A). The left pre‐pubic tendon was sutured to the pubis with simple interrupted sutures from the tendon to the predrilled holes in the left pubis. The wound on the vulva was closed with single interrupted monofilament sutures (Monosyn 0, B Braun, Rubi, Spain). Before closure of the laparotomy wound, a paramedial 1–1.5 cm incision in the abdominal wall (avoiding mammary gland damage) was performed to provide access for pre‐pubic urethrostomy, 2–3 cm cranially to the symphysis, and 3–4 cm laterally from the midline. After the proximal part of the urethra was passed through the incision and sewn to the skin using simple interrupted sutures (Dafilon 4–0, B Braun, Rubi, Spain) (Figure 2B), the laparotomy wound was closed in layers with a continuous suture of absorbable monofilament material (Monosyn 0, B Braun, Rubi, Spain). The skin along the entire length of the surgical wound was closed with single vertical mattress sutures of non‐absorbable material (Dafilon 0, B Braun, Rubi, Spain) (Figure 2C).

### 2.2 Hybrid surgery

The cadavers were placed in dorsal recumbency (Trendelenburg position). For proper intraoperative visualization, the operators were positioned on both sides of the operating table with a laparoscopic column placed behind the caudal side of the cadaver. The laparoscopic procedure started with the introduction of three trocars of 5 mm in diameter. All endoscopic equipment used for the laparoscopic procedure involved a 5‐mm 30° scope and was acquired from the same manufacturer (Karl Storz SE & Co. KG; Tuttlingen, Germany).

The first trocar for laparoscopic optics was inserted using the Hasson method at the level of the umbilicus.^13^ After insufflation of the abdominal cavity (CO_2_ pressure of 8 mmHg), two consecutive trocars were inserted caudal‐laterally to the first optical trocar (Figure 2D) under the control of the endoscope.

**FIGURE 3:**
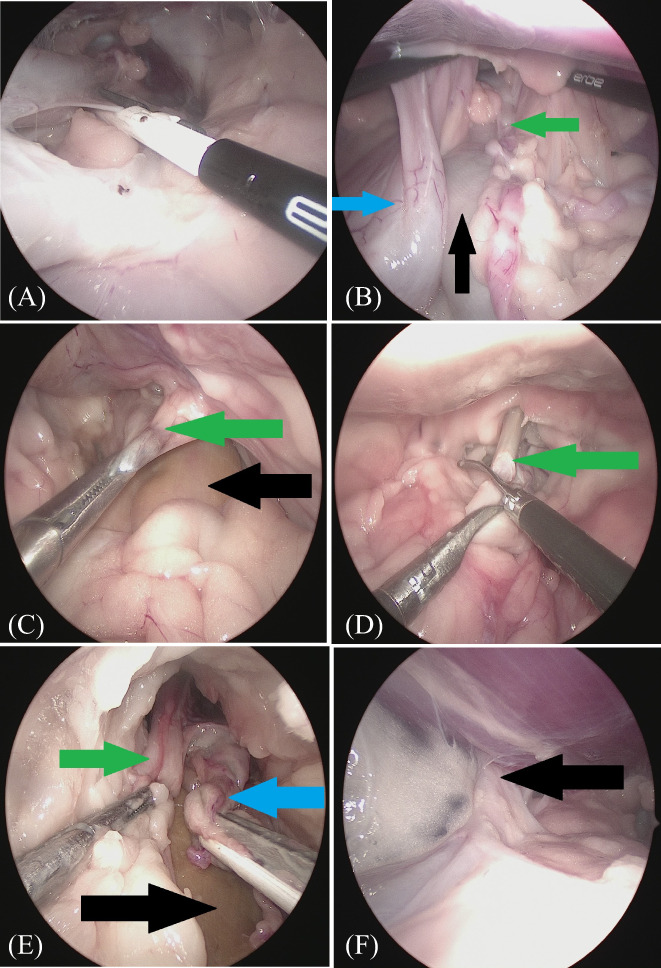
(A) Endoscopic intraoperative image of urethra dissection. (B) Endoscopic intraoperative image obtained after uterine dissection, showing the bladder (blue arrow), laparoscopically dissected body of the uterus (green arrow), and rectum (black arrow). (C) Intraoperative endoscopic image of the uterine stump (green arrow) before preparation in the hybrid spayed group (black arrow—rectum). (D) Intraoperative endoscopic image of the urethra cut transversely at half‐length (green arrow). (E) Endoscopic intraoperative image of the dissected urethra (green arrow) and the uterine stump (blue arrow) in the hybrid spayed group (black arrow—rectum). (F) Intraoperative endoscopic image of the urethrostomy site (black arrow) captured from the abdominal side

In the hybrid intact group, the procedure started with the removal of the right ovary and dissection of the same‐side broad ligament using a vessel‐sealing device (BiCision®, Erbe, Tübingen, Germany). An optical trocar was temporarily used opposite to the operated side of the working trocar to facilitate visualization during ovarian removal and dissection of the broad ligament of the uterus (Figure 2E). After completing these procedures on both sides, the uterine body was dissected up to half of the urethral length. The vesicogenital and pubovesical pouches were opened and dissected bluntly using laparoscopic forceps. The fatty tissue surrounding the urethra, uterine body and vagina was dissected and severed (Figure 3A,B).

**FIGURE 4:**
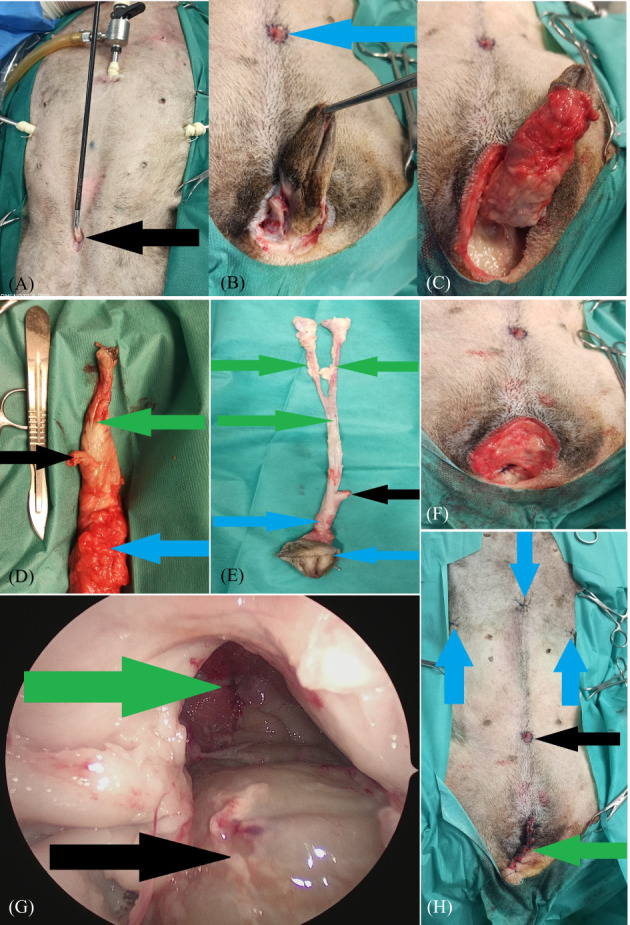
(A) Proximal part of the urethra was extracted through the abdominal wall incision during urethrostomy (black arrow). (B) Cutaneous incision line in vulvovaginectomy and the site of urethrostomy in the hybrid group (blue arrow). (C) Preparation of the vulva and vaginal vestibule in the hybrid group. (D) Urogenital organs removed from the hybrid spayed group, including the distal part of the urethra (black arrow), uterine stump (green arrow), and vulva and vaginal vestibule (blue arrow). (E) Urogenital organs removed from the hybrid intact group, including, distal part of the urethra (black arrow), body and horns of the uterus (green arrows), and vulva and vaginal vestibule (blue arrows). (F) Intraoperative external image of the wound during suturing after vulva removal. (G) Intraoperative endoscopic image of a sutured wound after removal of the urogenital organs through the vulvar incision, including anastomosis site (green arrow) and rectum (black arrow). (H) Postoperative wound in the hybrid group, including the wound after vulva resection (green arrow), trocar wounds (blue arrows), and pre‐pubic urethrostomy wound (black arrow)

In the hybrid‐spayed group, the procedure started with the dissection of the uterine stump to the same level as that in the hybrid intact group (Figure 3C). The remaining procedures were identical in both groups. After dissecting the distal part of the urethra, it was cut in the middle of its length with laparoscopic scissors (Figure 3D). The caudal part of the urethra along with the uterine body/stump was laparoscopically dissected as far caudally as intraoperatively possible (Figure 3E). The proximal part of the urethra was intraoperatively stitched to the edges of the skin wound. For this purpose, a surgical wound of 1–1.5 cm in length was made with a scalpel blade No. 11, at a site located 2–3 cm cranially to the pubic symphysis, and through the entire thickness of the abdominal wall at the level of the Linea alba. Laparoscopic forceps were inserted; under the control of the laparoscope, the proximal part of the urethra was grabbed and then passed through the pre‐pubic incision in the abdominal wall. The edges of the urethra were equalized with the use of surgical scissors and then sewn to the skin margins with single interrupted non‐absorbable material (Dafilon 4–0, B Braun, Rubi, Spain) (Figures 3F and 4A). The next stage involved dissection of the vulva and distal part of the urethra and vagina (part not previously dissected by laparoscopy) using open surgery. The technique proposed by Bilbrey et al.^14^ was used to perform vulvovaginectomy. A fusiform incision of the skin was made around the vulva. The deeper tissues were sharply dissected from the labia and vestibule. The constrictor vestibule and constrictor vulvae muscles were dissected from the vestibule. The dorsal labial branches of the ventral perineal artery were coagulated. The dissection was performed cranially around the vagina by transecting the ischiocavernosus and ischiourethralis muscles and passing cranially between the paired levator ani muscles to the level of the cervix. The vaginal branches of the vaginal artery and vein, as well as the uterine arteries and their branches to the vagina, were coagulated (Figure 4B,C). This procedure allowed for the final dissection of the vulva and vagina, along with the rest of the urethra. Separation of these organs from the surrounding tissues allowed them to be removed from the abdominal cavity and pelvis through a surgical wound made at the level of the vulva (Figure 4D,E). The wound at the level of the perineum was closed in layers after the removal of the vulva and vagina, starting with a single purse‐string suture inserted as deeply as possible into the pelvic lumen between the pelvic muscles and rectal wall. Subsequently, single interrupted sutures were placed on the muscles and subcutaneous tissue (Monosyn 0, B Braun, Rubi, Spain) (Figure 4F,G). The skin wound was sealed with single vertical mattress sutures. After performing open surgery and re‐obtaining pneumoperitoneum, the surgical field was assessed laparoscopically, and the trocars were removed. The trocar wounds were closed through all layers of the abdominal wall with single interrupted sutures of non‐resorbable material (Dafilon 0, B Braun, Rubi, Spain) (Figure 4H).

### 2.3 Surgical time and length of postoperative wounds

The duration of surgery in all groups was measured from the first skin incision to the last skin suture. The postoperative wound length was measured after the surgical procedure was completed, and the continuity of the rectal wall was assessed before the ureter leakage was tested. The length of the wound in the open technique group was measured using a standard tape measure, while that in the hybrid technique group was measured as the sum of the wound length at the level of the removed vulva and trocar wounds. The lengths of the post‐urethrostomy wounds were not measured, as they were similar in all groups.

### 2.4 Postoperative complications

**FIGURE 5:**
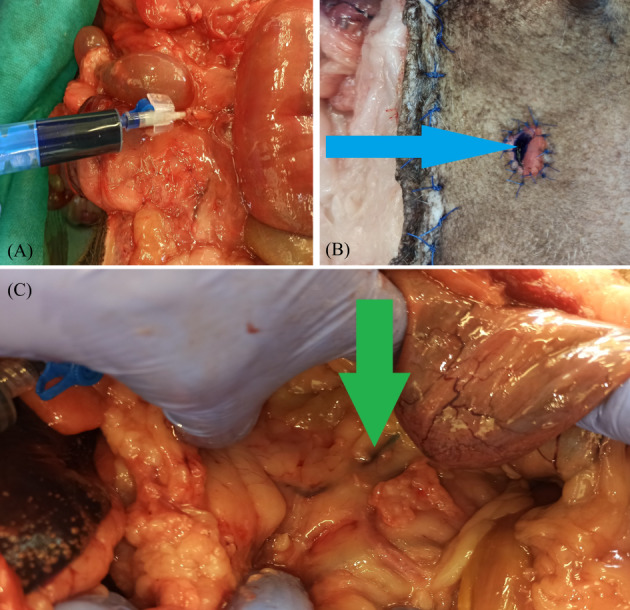
(A) Catheterization of the ureter at the level of the kidney was used to assess the patency and tightness of the ureters. (B) Presence of ink at the site of the urethrostomy suggests ureter is patent (blue arrow). (C) Ureteral patency and tightness were assessed using coloured ink filling (green arrow)

All ureters were post‐surgically tested for leaks. After the procedure was completed, the abdominal cavity was reopened (right paramedian access), and the ureters were severed at the level of the kidney. Subsequently, the lumen was catheterized using 22 G intravenous catheters (Figure 5A). To seal the catheter, a ligature made of 3–0 non‐absorbable monofilament material (Dafilon, B Braun, Rubi, Spain) was placed on the proximal part of the ureter after the catheter insertion. The next step in the leak assessment was the introduction of coloured ink through the inserted catheter (first blue to the right ureter, then black to the left ureter) until the ink appeared in the lumen of the sewn urethra during the experiment (Figure 5B). To assess tightness (no damage), the entire course of the ureters from the kidney to the bladder opening was macroscopically assessed (Figure 5C). The appearance of the rectum was also assessed macroscopically for any evidence of damage.

### 2.5 Statistical analyses

Descriptive statistics were estimated, and the normality of distribution assumption was tested with the Kolmogorov–Smirnov test with the Lilliefors correction. All variables were normally distributed (*p* > .05). Differences in the mean values of body weight, wound length and procedure duration were tested using one‐way analysis of variance (ANOVA) with Tukey's post‐hoc test. *p*‐values of <.05 were considered indicative of statistically significant findings. All analyses were performed using Statistica 13 (TIBCO Software Inc.).

## 3 RESULTS

The mean weight values of the cadaver dogs were 21.17 ± 7.98 kg, 31.00 ± 5.29 kg, 24.08 ± 8.52 kg and 25.25 ± 11.25 kg in the open intact, open spayed, hybrid intact, and hybrid spayed groups, respectively; these values were similar in all groups (*p* = .27).

The duration of surgery differed among the groups (*p* < .001). The surgical technique and reproductive status affected the duration of surgery, with the shortest and longest operative time estimates recorded for the open spayed (61.7 ± 4.8 min) and hybrid intact (91.0 ± 8.8 min) groups (*p* < .001). Significant differences in operative time were also observed between open spayed and open intact (*p* = .017), open intact and hybrid intact (*p* = .01) and hybrid spayed and hybrid intact (*p* < .001) groups. Surgical technique, but not reproductive status, affected wound length. The surgical wound was significantly longer in both open groups (26.08 ± 4.91 and 21.75 ± 2.38 cm in intact and spayed, respectively) than in both hybrid groups (9.00 ± 1.22 and 8.75 ± 1.04 cm in intact and spayed, respectively). However, operative time was similar between the groups within each operating technique category.

All surgical procedures were feasible, independently of dog breed or cadaver weight. No case of leakage or obstruction was observed. There was no damage to the rectum wall.

## 4 DISCUSSION

Neoplastic lesions of the urinary system account for <2% of all tumours observed in dogs, and their treatment remains a challenge.^1^, ^2^ Approach to lesions located in the distal part of the urethra, where organs are surrounded by pelvic bones is particularly challenging, and previously described methods have some limitations.^4^, ^5^, ^6^, ^8^, ^15^ Consequently, in the present study, we aimed to develop a novel surgical method applicable in this context and to test it on cadavers.

Indications for urethrostomy in companion animals include extensive injuries and neoplastic lesions of the distal urinary tract.^15^, ^16^ Feasible urethrostomy sites in female dogs include the abdominal wall,^15^ perineum,^14^ and vagina.^15^, ^16^ Suturing the urethra in a way that creates tissue tension can lead to postoperative complications.^14^, ^15^ Therefore, in cases of urethra length insufficient for perineal urethrostomy, pre‐pubic urethrostomy is recommended.^17^ In the present study, we sutured the urethra at the midline in the hybrid group because an extensive surgical wound was not present at this site; similar findings were observed in the classically operated group, affecting the performance of the urethrostomy lateral to the laparotomy line. Such urethrostomy sites are commonly used in companion animal surgery.^15^ Midline pre‐pubic urethrostomy is associated with a lower risk of urine leak onto the limb during voiding; therefore, some surgeons prefer to suture it to the midline laparotomy wound. In the group undergoing hybrid surgery, the authors performed urethrostomy with the assistance of laparoscopy, which allows the urethra to be sutured in a more favourable position. Queiroga et al.^18^ performed an experimental pre‐pubic urethrostomy assisted by laparoscopy in rabbits. A similar surgical procedure was described in a cat undergoing surgery due to recurrent feline lower urinary tract disease.^19^ In the present hybrid group, the urethra was prepared laparoscopically. This procedure may help ensure radical removal of the distal part of the urethra and reproductive organs under visual control. This approach may be used in cases of neoplastic tumours of the distal part of the urethra that require extensive resection, allowing healthy tissue margin to be preserved.

The lowest risk of postoperative urinary incontinence has been reported for the urethra resected between one‐third to half of its length measured from its distal part in humans^20^ and animals.^5^, ^14^ Using these indicators, White et al.^5^ performed vaginourethroplasty for the treatment of urethral obstruction in six sterilized female dogs, showing good long‐term outcomes in most cases. The proposed approach may enable radical removal of the target lesion and follow‐up assessments of tumour recurrence, supporting timely treatment.

Surgical approach to pelvic organs is challenging, and feasible options include access via the caudal abdomen,^21^ a bilateral pubic and ischial osteotomy,^12^, ^16^, ^22^ and sagittal pelvic osteotomy, among others.^5^, ^23^ Access via the caudal abdomen is less invasive than the other options; however, it is associated with limited intraoperative visibility and restricted access to the pelvic organs. Pelvic osteotomy may provide satisfactory intraoperative access and visibility; however, it increases the extent of trauma. The present study used bilateral pubic and ischial osteotomy proposed by Allen and Crowell,^12^ who previously achieved satisfactory intraoperative access to the pelvic cavity organs in dogs with this technique. The feasibility and outcomes of this classical technique were compared with those of the proposed hybrid surgical technique. Both approaches enable access to and control of the operative field. However, the hybrid method was associated with wounds that were smaller than those associated with the open method, suggesting an advantage of endoscopic surgery. In addition, osteotomy and osteosynthesis of the pelvis in the hybrid method are not associated with postoperative complications or pain. There was no difference in wound length between the open method groups.

The hybrid technique may be less invasive than the open technique. Previous studies have shown that endoscopic surgery has advantages over open surgery in some types of procedures.^24^, ^25^ However, some controversy remains, partly because of the multifactorial approach to post‐surgical assessments. Operative time, wound size, and the impact of CO_2_ on the operated organism (endoscopic surgery) may determine the suitability of each technique.^25^ The reproductive status of the study dogs affected operating time; regardless of surgery type, operative time was reduced for spayed dogs. However, the mean duration of surgery in dogs with the same reproductive status (open spayed vs. hybrid spayed and open intact vs. hybrid intact) was shorter in the open group than in the hybrid group; these findings are consistent with those of previous studies.^26^ Nevertheless, data on hybrid surgery duration are lacking, making comparisons difficult; in addition, the present study involved cadavers, precluding conclusions about operative time in living dogs.

Ureteral injuries are serious complications that may occur in this context.^27^, ^28^, ^29^, ^30^ No case of ureteral wall damage was observed in the present study; however, particular care should be taken to prevent this type of damage in living animals undergoing surgery. Ovariohysterectomy is among the most common procedures performed on companion animals.^31^, ^32^, ^33^, ^34^ Several relevant laparoscopic techniques have been described,^24^, ^32^, ^35^ including insertion of an optical trocar into the linea alba at the umbilicus level and that of two working trocars lateral to the optical trocar,^35^ as used in the present study. The present findings suggest that the proposed hybrid and open methods for the surgical treatment of tumours in the distal part of the urethra are technically feasible.

This study has some limitations. First, the present study involved dog cadavers, precluding meaningful discussion about intraoperative difficulties associated with operating on live dogs. Second, the use of cadavers in this study removed the opportunity for short‐ and long‐term follow‐up assessments. Third, all study procedures were performed on unchanged urogenital organs; consequently, it remains unclear whether a healthy tissue margin can be maintained during the removal of neoplastic lesions in all cases. Nevertheless, this was a pilot study whose aim was to evaluate the feasibility of the proposed technique; this study has achieved its aim.

## 5 CONCLUSION

The proposed open and hybrid methods for the surgical treatment of urethral tumours are technically feasible. These procedures may be considered in animals ineligible for other less extensive procedures. In vivo experimental studies are required before the proposed techniques may enter veterinary practice.

## CONFLICT OF INTEREST

The authors declared no potential conflicts of interest with respect to the research, authorship and/or publication of this article.

## Data Availability

All data of this study are available in the manuscript.
